# Massive Myocardial Calcification

**DOI:** 10.1016/j.jaccas.2025.103384

**Published:** 2025-04-02

**Authors:** Mehran Babady, Charalampia Maltsinioti, Ermal Limaj, Oliver Bruder, Christina Karamarkou

**Affiliations:** Department of Cardiology and Angiology, Elisabeth Hospital Essen, Essen, Germany

**Keywords:** cardiomyopathy, computed tomography, disorders of calcium metabolism, echocardiography, pulmonary hypertension

## Abstract

**Background:**

Massive myocardial calcification is an extremely rare condition with diverse etiologies, often requiring multimodality imaging for diagnosis.

**Case Summary:**

A 73-year-old woman with coronary artery disease and a history of ovarian carcinoma presented with dizziness and dyspnea. Echocardiography revealed a large floating structure on the anterior mitral leaflet, left ventricular hypertrophy, and extensive myocardial calcification. Preoperative imaging confirmed calcifications, and surgery included decalcification, subvalvular myectomy, and aortic valve replacement. Histology revealed fibrosis and scarring. Four years later, she developed pulmonary hypertension, increased calcification, and right ventricular dysfunction. Despite thorough evaluation, the etiology of the calcification remained unclear, with hypertrophic cardiomyopathy considered a probable underlying condition.

**Discussion:**

This case highlights a rare presentation of myocardial calcification, emphasizing the value of multimodality imaging and comprehensive diagnostic workup. It contributes to the limited literature on the condition and its management challenges.

**Take-Home Messages:**

Multimodality imaging and a systematic approach are vital for diagnosing and managing rare cardiac conditions like massive myocardial calcification.

## Graphical Abstract

**Figure:**
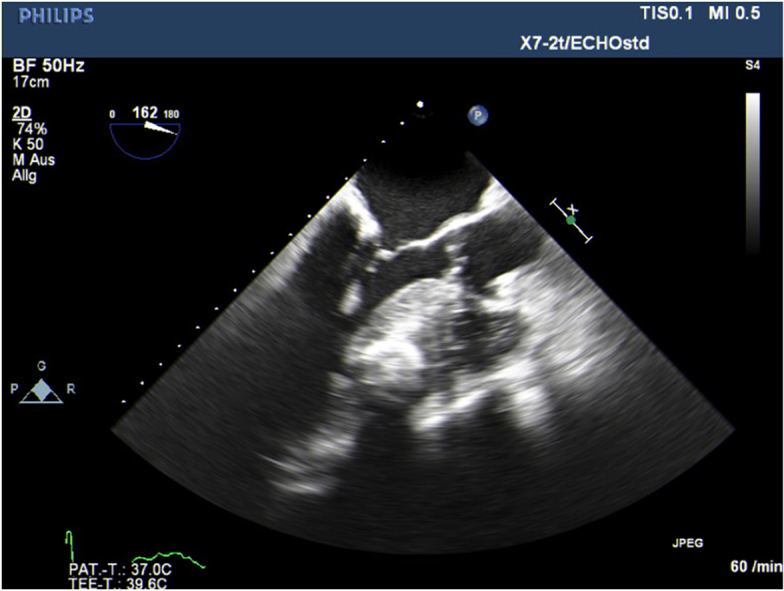

## History of Presentation

**Figure 1:**
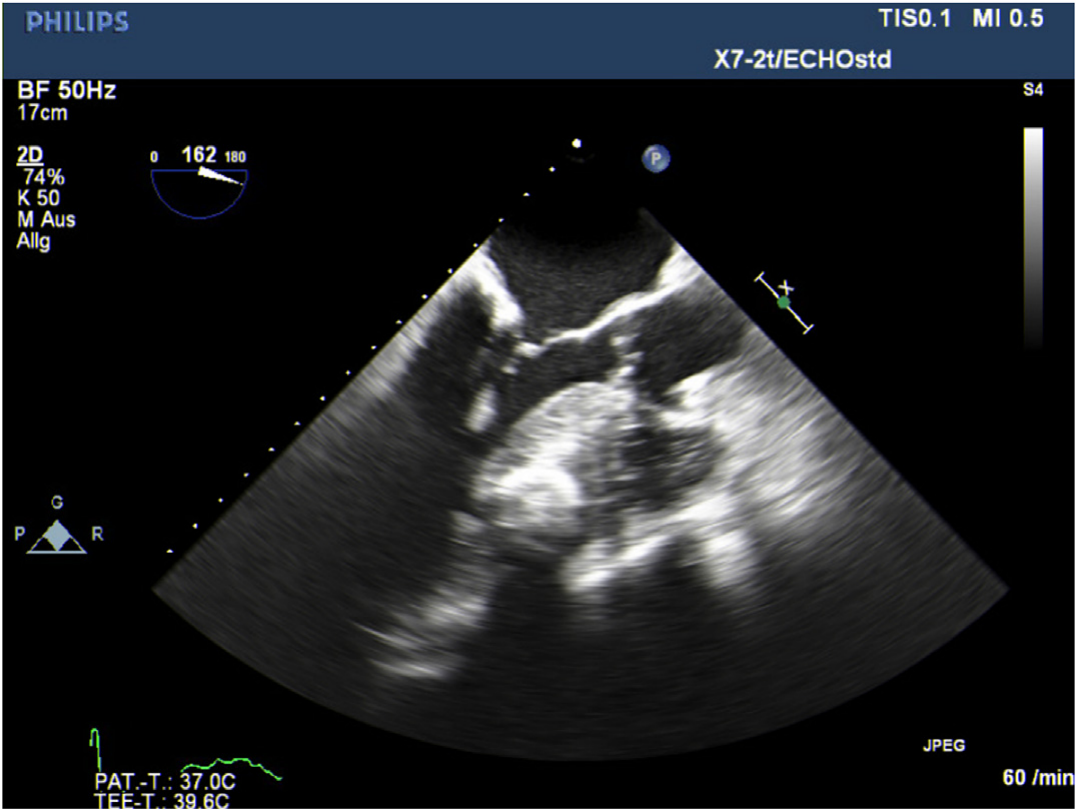
Echocardiographic Findings of Massive Myocardial Calcification Transesophageal echocardiography showing a significant septal left ventricular hypertrophy with calcified myocardium and a vegetation on the posterior mitral leaflet.

We report a case of a 73-year-old woman who was referred to us from an outside hospital because of progressive dizziness and exertional dyspnea. An echocardiogram revealed a large floating structure (27 × 17 mm) attached to the anterior mitral leaflet along with hypertrophy and massive calcification of the left ventricle. Notably, significant involvement of the midventricular septum was observed, leading to subvalvular left ventricular outflow tract (LVOT) obstruction (Figure 1). On explicit inquiry, the patient reported experiencing breathing difficulties during exertion in her youth. According to the patient, an ultrasound examination at that time revealed that her heart was enlarged.

**Take-Home Messages**

• Massive myocardial calcification is a rare condition with multiple possible etiologies.
• This case highlights the importance of early identification, thorough investigation of potential causes, and effective management of contributing factors while emphasizing the critical role of multimodality imaging in diagnosis and follow-up.

## Medical History

The patient was known for coronary artery disease and had a history of ovarian carcinoma. She had undergone an adnexectomy, pelvic lymphadenectomy, and adjuvant chemotherapy with carboplatin and paclitaxel.

## Differential Diagnosis

Massive myocardial calcification is a very rare condition that manifests because of a range of different etiologies (Table 1). In terms of cause, they are classified into metastatic and dystrophic.^1^ Metastatic calcification occurs as a result of elevated serum calcium levels and most commonly affects patients with chronic renal failure undergoing hemodialysis.^2^^,^^3^ Additionally, hyperparathyroidism, calcium and vitamin D deficiency, and oxalosis can also contribute to the development of myocardial calcifications. Dystrophic calcification arises from calcium deposits forming in necrotic tissue, which can be caused by a range of myocardial injuries such as myocarditis, myocardial infarctions, tuberculosis, sarcoidosis, hemorrhage, endomyocardial fibrosis (EMF), and idiopathic mitral annular calcification (MAC).^2^^,^^4^

**Table 1:** Etiologies of Massive Myocardial Calcification^2^

Metastatic
Calcium or vitamin D deficiency Hyperparathyroidsim Oxaluria Renal failure
Dystrophic
Drug-induced (eg, steroids, cyclosporine) Infectious (myocarditis, tuberculosis) Inflammatory (sarcoidosis, rheumatic heart disease, sepsis, endomyocardial fibrosis) Ischemic (myocardial infarction) Mitral annular calcification Neoplastic Traumatic (cardiac surgery, hemorrhage)
Idiopathic

## Investigations

In regards of the floating mitral valve structure morphologically, there was suspicion of a fibroelastoma or a vegetation, although clinically and microbiologically no evidence of an endocarditis was found. Because of the high risk of embolization in either case and because of the presence of hypertrophy-related LVOT obstruction as well as significant aortic valve stenosis, we decided to proceed with surgery for the patient.

**Figure 2:**
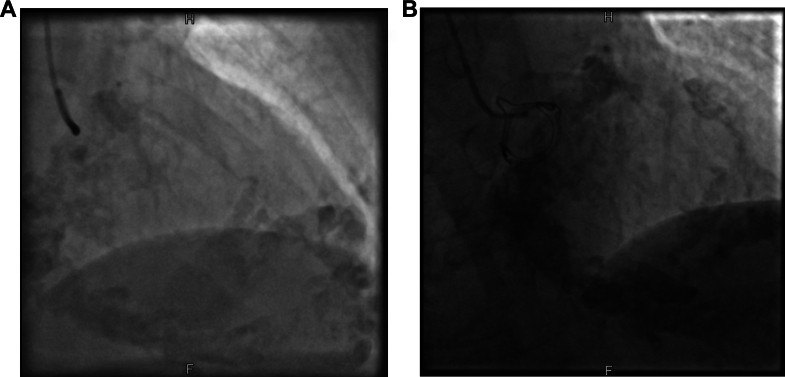
Fluoroscopy Depicting Myocardial Calcification Fluoroscopy as part of a cardiac catheterization showing massive myocardial calcification in the right anterior oblique projection at a 30° and a cranial projection at a 30° (A) and the progression within 4 years (B).

**Figure 3:**
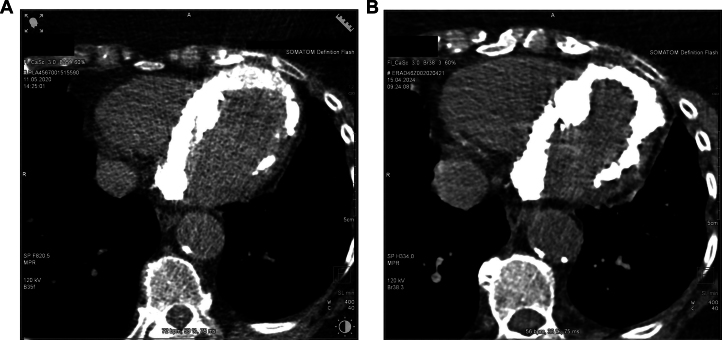
Cardiac Computed Tomography of Myocardial Calcification Cardiac computed tomography showing massive myocardial calcification of the left ventricle (A) and the progression within 4 years (B).

Preoperatively, coronary angiography revealed no significant coronary stenoses in terms of the known coronary artery disease. However, during the examination, significant myocardial calcification observed echocardiographically was confirmed (Figure 2A). Additionally, computed tomography (CT) of the heart was performed, revealing excessive calcifications of the left ventricular myocardium, with emphasis on the septum and apex. Furthermore, there were significant calcifications of the mitral valve annulus suggestive of caseous calcification of the mitral annulus (Figure 3A).

## Management

During the intraoperative procedure, removal of the floating structure, decalcification of the anterior mitral leaflet, subvalvular myectomy with excision of a hypertrophic myocardial portion next to the calcification, and biological aortic valve replacement (BAVR) due to an aortic valve stenosis were performed. Histologic examination of the intraoperatively removed floating mitral valve structure revealed fibrin components, enclosed basophil granular structures, nucleated cells, and neutrophilic granulocytes, consistent with endocarditis.

Regarding the myocardial calcification of the midventricular septum, histologic examination of the hypertrophed myocardium revealed endocardium and myocardium with significant scarring (which could be well visualized with Elastika-van-Gieson staining), intramural blood vessels without abnormalities, no inflammation, and cardiomyocytes with slight variation in nuclear size, indicating evidence of EMF and interstitial fibrosis.

Postoperatively, the patient was readmitted to our cardiology clinic. We continued the antibiotic endocarditis therapy in accordance with guidelines alongside postoperative care and discharged the patient in good clinical condition thereafter.

## Outcome and Follow-Up

Four years later, the patient was admitted to our hospital by her cardiologist. She presented again with increasing exertional dyspnea. On the transthoracic echocardiogram, we now observed an increasing left ventricular hypertrophy with a preserved left ventricular systolic function and a diastolic dysfunction. However, there was no gradient increase in the LVOT under Valsalva maneuver. In addition, we noticed signs of pulmonary hypertension with a systolic pulmonary artery pressure of 99 mm Hg. Furthermore, during a magnetic resonance imaging (MRI) examination, right ventricular dilatation and impaired right ventricular function with a right ventricular ejection fraction of 29% were confirmed as a consequence of pulmonary hypertension. A follow-up CT of the heart revealed a significant increase in myocardial calcification (Figure 3B).

**Figure 4:**
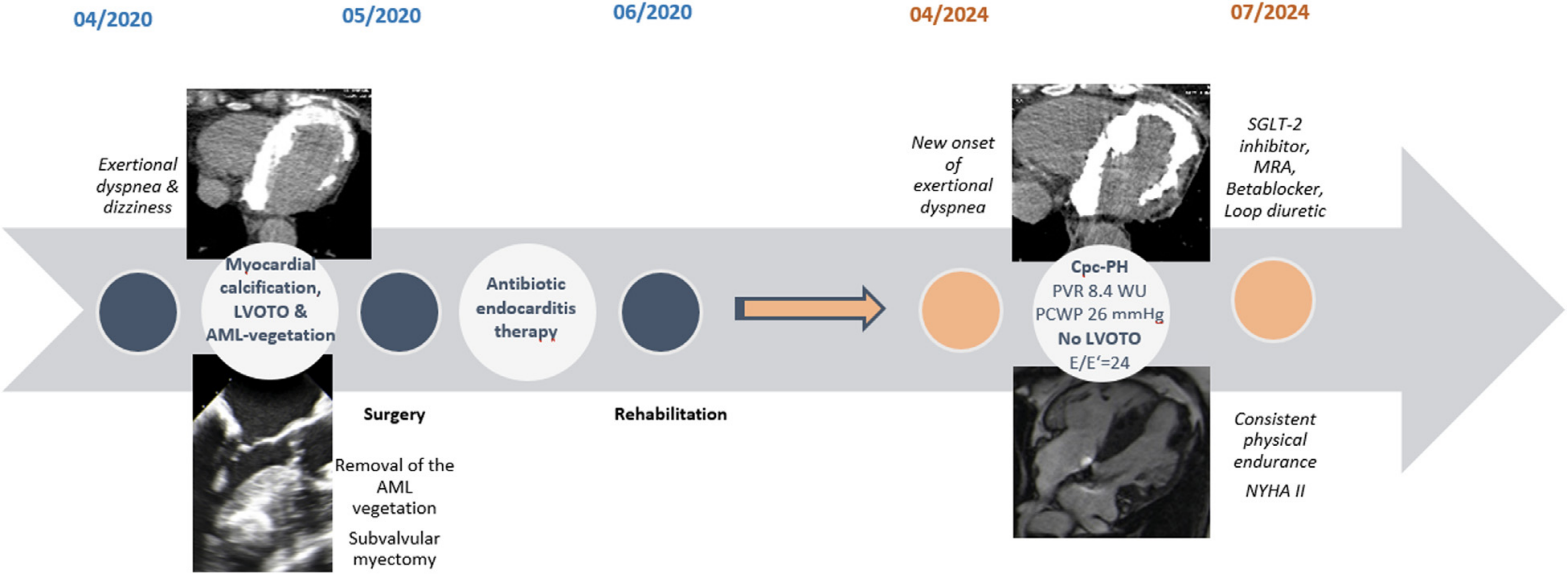
Clinical Timeline of Massive Myocardial Calcification Progression Clinical timeline illustrating the progression of symptoms, diagnostic findings, treatment interventions, and outcomes in a patient with massive myocardial calcification. AML = anterior mitral leaflet; Cpc-PH = combined precapillary and postcapillary pulmonary hypertension; E/Eʹ = ratio of early mitral inflow velocity to mitral annular early diastolic velocity; LVOTO = left ventricular outflow tract obstruction; MRA = mineralocorticoid receptor antagonist; PCWP = pulmonary capillary wedge pressure; PVR = pulmonary vascular resistance; SGLT-2 = sodium-glucose cotransporter-2; WU = Wood unit.

The patient also underwent cardiac catheterization. Coronary angiography ruled out progression of her known coronary artery disease, and right-sided cardiac pressures and resistances were determined during a right heart catheterization. During this procedure, the diagnosis of combined precapillary and postcapillary pulmonary hypertension was established, with a mean pulmonary artery pressure of 58 mm Hg, pulmonary capillary wedge pressure of 26 mm Hg, and pulmonary vascular resistance of 8.4 WU (Figure 4). In the clinical context, we interpreted the combined precapillary and postcapillary pulmonary hypertension to be caused by the chronic severe diastolic dysfunction because of massive myocardial calcification of unclear etiology.

## Discussion

The cause of the development of massive myocardial calcification in our patient remains unclear. Nevertheless, various hypotheses could be considered (Table 1). The rare occurrence of a metastatic etiology of myocardial calcification could be excluded easily through laboratory diagnostics by focusing on calcium metabolism.^1^ Serum calcium and phosphate levels, as potential indicators of disrupted calcium homeostasis, were within normal ranges. Additionally, renal retention values were normal, there was no evidence of hyperparathyroidism, and vitamin D levels were unremarkable as well. Furthermore, a urinalysis for oxalate was conducted, which also excluded oxaluria as a cause of myocardial calcification, as previously described in case reports. Additionally, the patient denied specific dietary habits that promote oxaluria, such as the excessive consumption of rhubarb, beetroot, spinach, or celery.^5^

The second and more commonly occurring dystrophic cause of massive myocardial calcification now seemed more likely in our patient. A possible cause for a dystrophic etiology might be MAC, which presented on cardiac CT alongside the massive myocardial calcification (Figures 3A and 3B).^6^ Regarding the distribution of MAC, a predominant involvement of the posterior mitral leaflet was observed. However, a predominant anteroseptal calcification of the myocardium with minimal contact to the mitral annulus rather argues against MAC as the cause of the massive myocardial calcification.

Another possible cause for myocardial calcification is EMF, which involves fibrosis of the endocardium and sometimes the myocardium. EMF can also be associated with myocardial calcification.^7^ During the histologic examination of the perioperatively removed myocardium, EMF was observed. However, this finding is nonspecific and may be due to necrosis, because there was no histologic evidence of eosinophilia. Additionally, echocardiography revealed evidence of left ventricular restriction with a ratio of early mitral inflow velocity to mitral annular early diastolic velocity of 24, which could be caused by EMF but could also be explained by the myocardial calcification. Cardiac MRI, on the other hand, did not suggest EMF. Furthermore, our patient did not have a history of staying in tropical or subtropical regions, which is typical for EMF.

Regarding the patient's previous chemotherapy for ovarian carcinoma, a drug-induced toxic cause of the massive myocardial calcification should also be considered. Cases of chemotherapy-associated myocardial infarctions have been described in the literature for both paclitaxel and cisplatin.^8^ However, we did not find any cases of massive myocardial calcifications associated with these chemotherapeutic agents in our research.

After a more detailed medical history, the patient reported reduced physical capacity during childhood and an ultrasound finding of heart wall thickening at age 16. Given the asymmetrical and likely progressive left ventricular hypertrophy detected by echocardiography during the initial admission, a possible hypertrophic cardiomyopathy (HCM) should be considered as the underlying cause of the myocardial calcification in the differential diagnosis. The emphasized involvement of the myocardial right ventricular insertion points in the calcification observed on CT would be indicative of a predominant calcification process consistent with a typical MRI late gadolinium enhancement pattern in HCM.

Regarding a possible autoimmune cause of the massive myocardial calcification, such as in the context of scleroderma, all laboratory diagnostics including Scl-70, CENP A, CENP B, RP11, RP155, fibrillarin, NOR90, Th/To, PM-Scl100, PM-Scl75, Ku, PDGFR, and Ro-52 were unremarkable, and the ACR/EULAR score was low. Additionally, a negative Quantiferon test was observed, which excluded tuberculosis as a cause for the calcification. In terms of sarcoidosis as a possible origin, there were also unremarkable laboratory findings, including a normal soluble interleukin-2 receptor and angiotensin-converting enzyme levels. Radiologically, no sarcoidosis-typical extracardiac findings were observed. Furthermore, the patient denied a history of rheumatic fever or myocarditis. Either of these conditions can also be causative factors for myocardial calcification.^4^

## Conclusions

The cause of the massive myocardial calcification in our patient remains unclear. However, HCM, which is today medically treatable in its early stages, appears to be a probable underlying predisposing condition in this patient‘s clinical context.

## Funding Support and Author Disclosures

The authors have reported that they have no relationships relevant to the contents of this paper to disclose.
